# Asymmetric Directional Multicast for Capillary Machine-to-Machine Using mmWave Communications

**DOI:** 10.3390/s16040515

**Published:** 2016-04-11

**Authors:** Jung-Hyok Kwon, Eui-Jik Kim

**Affiliations:** Department of Convergence Software, Hallym University, 1 Hallymdaehak-gil, Chuncheon-si, Gangwon-do 24252, Korea; jhkwon@hallym.ac.kr

**Keywords:** asymmetric sectorization, capillary machine-to-machine, directional multicast, Internet of things, millimeter Wave communications

## Abstract

The huge demand for high data rate machine-to-machine (M2M) services has led to the use of millimeter Wave (mmWave) band communications with support for a multi-Gbps data rate through the use of directional antennas. However, unnecessary sector switching in multicast transmissions with directional antennas results in a long delay, and consequently a low throughput. We propose asymmetric directional multicast (ADM) for capillary M2M to address this problem in mmWave communications. ADM provides asymmetric sectorization that is optimized for the irregular deployment pattern of mulicast group members. In ADM, an M2M gateway builds up asymmetric sectors with a beamwidth of a different size to cover all multicast group members with the minimum number of directional transmissions. The performance of ADM under various simulation environments is evaluated through a comparison with legacy mmWave multicast. The results of the simulation indicate that ADM achieves a better performance in terms of the transmission sectors, the transmission time, and the aggregate throughput when compared with the legacy multicast method.

## 1. Introduction

Capillary machine-to-machine (M2M) refers to networking technologies that provide devices with wireless connectivity in a local area by using various communications standards, including Bluetooth Low Energy (BLE), ZigBee, Wi-Fi, and WiGig [1,2]. M2M can be implemented in various intelligent services, such as home automation, smart healthcare, connected automobiles, and smart manufacturing. Therefore, there is consensus in the industry and academia that capillary M2M is a key enabler to realize the Internet of things (IoT) [3,4,5,6]. Traditionally, M2M services have been limited to low data rate applications that use wireless sensor networks (WSNs) [7,8]. However, the scope of their use has progressively extended to higher data rate applications due to the increasing demand for real-time streaming of high-definition television (HDTV), high-speed data transfer, and wireless 3D gaming. This trend has led to the adoption of millimeter Wave (mmWave) band communication technologies like IEEE 802.11ad, ECMA-387, and IEEE 802.15.3c, in M2M services [9,10,11].

mmWave band communication can support a multi-Gbps data rate with directional antennas, and it can be used for various M2M services, particularly for high data rate indoor services such as ad hoc conferences, smart classrooms, and telecasts [12,13]. In these applications, the devices that users have can be divided into multiple service groups, and each group might request different multimedia contents such as video streaming, presentation material from a dedicated local server (e.g., projector, presenter’s laptop). The local server should support a sufficient data rate to multiple groups via a ‘group-to-project connection’, for which mmWave multicast transmission is an indispensable technology for creation of the seamless interaction service [14,15,16]. In the mmWave band, the use of directional antennas is considered essential even for multicast transmission due to the short propagation distance problem caused by the unique characteristics of mmWave frequency band such as its oxygen absorption and high path loss [17,18,19]. Directional antennas form a narrow fan-shape beam in a particular direction, and thus the use of these makes it difficult to cover the entire multicast group consisting of members that are randomly placed around the sender. For multicast transmission with directional antennas, switching the beam direction with a fixed beamwidth in a clockwise or anticlockwise manner has been generally considered [20]. However, this approach may cause excessive power consumption and a long delay. Therefore, the appropriate tuning of the beamwidth and beam direction for multicast transmission is a challenging issue for mmWave band communication [21,22].

Several studies have been carried out to efficiently transmit multicast data with directional antennas. Guo *et al*. [23] proposed multicast transmission with multiple beam antennas. This method simultaneously covers multicast devices that are scattered around the sender, but it suffers from the high complexity of beamforming and the high cost of installing multiple beam antennas. A one-hop relay transmission for multicast transmission with a directional antenna was proposed in [24,25]. This method effectively extends the transmission range without adjusting the beamwidth. However, the overall network throughput is degraded due to the high overhead of the relay transmission. Sen *et al*. [26] proposed a priority-based multicast transmission named ‘beamcast’ in which a sender first transmits packets to nearby multicast devices by using omnidirectional antennas and then transmits packets to the rest of multicast devices using directional antennas. However, this approach exhibits a high performance only when multicast devices are near the sender while unnecessary sectors and duplicate packet reception may also occur.

In this paper, we propose an asymmetric directional multicast transmission (ADM) for a capillary M2M system that uses the minimum number of sectors with different beamwidth sizes to cover multicast devices deployed in a given service area. The operation of the ADM consists of three main phases: *Device discovery*, *Coverage region* (*CR*) *allocation*, and *Asymmetric sectorization*. In the device discovery phase, the M2M gateway (GW) discovers multicast group members by using the beamwidth with the smallest size, and it then obtains their sector IDs and locations. Next, during the CR allocation phase, the M2M GW allocates a CR value to each sector by considering the maximum distance of the multicast group member. The CR is a partitioned region within a given sector coverage in which the boundaries have been pre-determined based on a set of transmission ranges of M2M GW. The CR value of the initial sector is determined as the CR ID of the CR for which the farthest multicast group member is located. During asymmetric sectorization, the M2M GW decides the beamwidth size of the new sectors by referring to the allocated CR, and it announces the updated sector information to its multicast group members. An experimental simulation verifies that the ADM can improve the average system throughput by approximately 31% and can reduce the average transmission time by approximately 20% when compared to the legacy mmWave multicast approach.

The rest of this paper is organized as follows. Section 2 provides the system model that includes the directional antenna and the mmWave wireless personal area network (WPAN). The detailed operation of the ADM is described in Section 3. An experimental simulation is conducted in Section 4, and Section 5 concludes this paper.

## 2. System Model

### 2.1. Directional Antenna

To ensure backwards compatibility and cost efficiency, we consider a conventional sector antenna array instead of an adaptive antenna array. A sector antenna array consists of a number of fixed beams, and one or more beams are combined to form a sector that corresponds to the transmission coverage of the sender. We assume that the direction and the beamwidth of the sector are determined according to the beam forming codebook defined in the IEEE 802.15.3c standard [11]. For simplicity of the planar analysis for the mmWave band communication, we assume that all M2M devices exist in the same plane and use a two-dimensional flat-top directional antenna.

**Figure 1:**
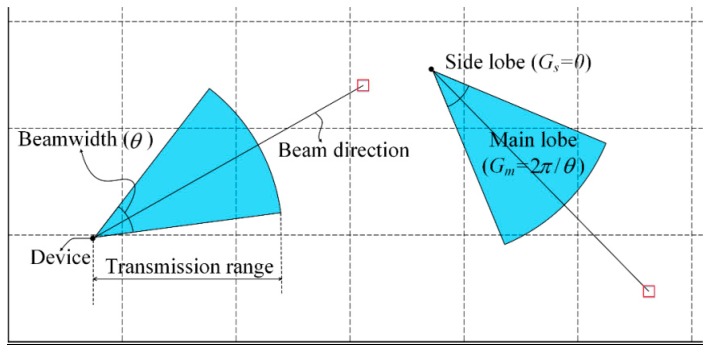
Two-dimensional flat-top directional antenna model.

Figure 1 shows an example of the two-dimensional flat-top directional antenna model. This model has a constant antenna gain only within the beamwidth, and the antenna gains of the main lobe ($G_{m}$) and the side lobe ($G_{s}$) are ‘2π/beamwidth’ and ‘0’, respectively. Considering that the beamwidth of each beam is θ, the beamwidth of a sector can be chosen from θ to 2π in θ units. Therefore, the maximum number of sectors is ‘2π/θ’ with a minimum number of sectors of ‘1’. Meanwhile, the maximum transmission range of the antenna is represented as TR, which can be calculated using Equation (1).

(1) $$\text{TR}=10^{\frac{\kappa }{10n}}\text{ , }(\kappa =P_{t}+G_{t}+G_{r}-I_{L}-PL_{0}-\gamma_{th})$$

where $P_{t}$ is the transmission power, $G_{t}$ and $G_{r}$ are the transmitter and receiver antenna gains, $I_{L}$ is the implementation loss, $PL_{0}$ is the path loss at 1 m, $\gamma_{th}$ is the receiver sensitivity, and n is the path loss exponent. Table 1 lists an example of the maximum transmission range of the directional antennas for varying beamwidths and the modulation and coding schemes (MCSs). The beamwidth size is smaller and the transmission range is longer because the sector with a small beamwidth has a high antenna gain. Moreover, the transmission range varies according to the MCS, which affects the receiver sensitivity of the M2M devices.

### 2.2. Network Model

We consider a centralized network model where a number of M2M devices form a capillary M2M and one of those becomes an M2M GW that acts as a personal area network coordinator (PNC). The M2M GW performs various procedures including neighbor discovery, synchronization, channel time scheduling, and network termination to manage the overall capillary M2M network. For multicast transmission with a directional antenna, the M2M GW manages the multicast group members, announces multicast information and assigns the channel time. Moreover, it transmits multicast packets by switching the direction of the antennas. Considering that all M2M devices use a directional antenna, M2M GW should select a proper beamwidth size for the sector and should schedule a sector switching timing to cover all sectors.

In this paper, we consider the single carrier mode of mmWave physical layer (SC-PHY) specified in the IEEE 802.15.3c WPAN standard [11,27]. SC-PHY is designed to support the low complexity and the high energy-efficiency, and specifies three classes for 14 MCSs, which are listed in Table 2. Thus, according to their supported data rates, the diverse mmWave applications can be served. The SC-PHY frame includes the PHY preamble, the frame header, and the payload. The PHY preamble prior to the frame header supports frame detection, frame synchronization, and channel estimation. The frame header includes PHY header, medium access control (MAC) header, and header check sequence (HCS) for the frame decoding. Finally, the payload includes the data, and its maximum length is supported up to 1 Mbytes.

## 3. Design of the ADM

In the transmission with directional antennas, the M2M GW should repeat the transmission for the same packet as the number of formed sectors in order to cover all multicast group members. This causes the degradation of the network performance in terms of the throughput and delay. Therefore, it is necessary to minimize the redundant multicast transmissions by reducing the number of sectors within the capillary M2M network. The ADM is designed to build up an optimal number of asymmetric sectors for directional multicast transmissions by taking the distribution of M2M devices into account. Note that ‘asymmetric’ means that each sector could maintain a beamwidth of a different size with the corresponding difference in the transmission range. In this section, we describe the design of the ADM in detail. As mentioned earlier, the operation of the ADM consists of three phases: (1) Device discovery, (2) CR allocation, and (3) Asymmetric sectorization.

During device discovery, the capillary M2M network operates over the initialized ‘symmetric’ sectors that have a unit beamwidth. Hereafter, this is referred to as the unit-beam sector. The M2M GW broadcasts multicast information via a beacon for all unit-beam sectors, and each multicast group member responds with a join request message. From the join requests, the M2M GW obtains the information for multicast group members, including the device ID, current sector ID, and distance to the M2M GW. The distance to each M2M device from the M2M GW can be represented using the matrix $D_{\left(i\right)}$, as in Equation (2).

(2) $$D_{\left(i\right)}=\left[d_{\left(i,1\right)},d_{\left(i,2\right)},\cdot \cdot \cdot ,d_{\left(i,j\right)},\cdot \cdot \cdot ,d_{\left(i,N_{i}\right)}\right],\;0<i\leq M,0<j\leq N_{i}$$

where *i* is a unit-beam sector ID, *j* is an M2M device ID within the unit-beam sector *i*, *M* is the total number of unit-beam sectors, $N_{i}$ is the number of M2M devices within the unit-beam sector *i*, and $d_{\left(i,j\right)}$ is the distance value between the M2M GW and device *j* within the unit-beam sector *i*.

**Figure 2:**
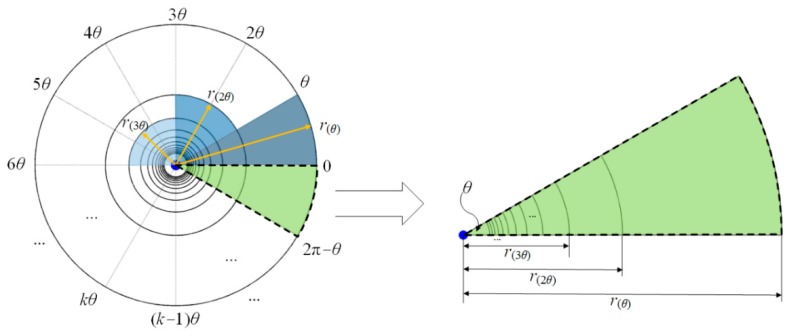
Coverage region.

During the CR allocation phase, the M2M GW allocates a CR value to each unit-beam sector. Figure 2 describes the structure of the CR. In the figure, the unit-beam sector is virtually divided into multiple CRs with a unique CR ID, k, and their boundaries are defined as the transmission ranges for varying beamwidths (refer to Table 1.). In other words, the CR is a partitioned region within the coverage of a unit-beam sector, and its area is pre-determined based on a set of transmission ranges of the M2M GW, which is represented by the matrix R, as in Equation (3).

(3) $$R=\left[r_{\left(\theta \right)},r_{\left(2\theta \right)},\cdot \cdot \cdot ,r_{\left(k\theta \right)},\cdot \cdot \cdot ,r_{\left(2\pi \right)}\right],\;0<k\leq 2\pi \theta^{-1}$$

where θ is a beamwidth of unit-beam sector, k is a CR ID, and $r_{\left(k\theta \right)}$ is a CR boundary of ${\text{CR}}_{k}$, which is the same as the transmission range of the M2M GW when the beamwidth size is kθ.

The CR value of unit-beam sector is determined as the CR ID of CR where the farthest M2M device is located. Specifically, to allocate a CR value, the M2M GW searches the maximum distance, $\max(d_{\left(i,j\right)})$ within the matrix $D_{\left(i\right)}$, and then the CR value of the unit-beam sector i, $cr_{\left(i\right)}$ is determined as k (*i.e.*, the CR ID of CR containing the device with $\max(d_{\left(i,j\right)})$), as follows.

(4) $$cr_{\left(i\right)}=\left\{ \begin{array}{l} k,\;r_{\left((k+1)\theta \right)}\leq \max(d_{\left(i,j\right)})<r_{\left(k\theta \right)}\\ 0,\;D_{\left(i\right)}=\phi \end{array}\right.$$

Note that, if there is no device within a unit-beam sector, the M2M GW sets the CR value to zero. The CR value of the each unit-beam sector is represented by the matrix CR, as in Equation (5).

(5) $$CR=\left[cr_{\left(1\right)},cr_{\left(2\right)},\cdot \cdot \cdot ,cr_{\left(i\right)},\cdot \cdot \cdot ,cr_{\left(M\right)}\right],\;0<i\leq M$$

In the asymmetric sectorization phase, the M2M GW conducts an asymmetric sectorization procedure where the sectors of the directional antenna are newly configured, and each sector (which is hereafter referred to as an asymmetric sector) can maintain different beamwidth size and obtain a newly allocated unique sector ID. The result of the asymmetric sectorization procedure is represented as the matrix AS, as in Equation (6).

(6) $$AS=\left[as_{\left(1\right)},as_{\left(2\right)},\cdot \cdot \cdot ,as_{\left(i\right)},\cdot \cdot \cdot ,as_{\left(M\right)}\right],\;0<i\leq M$$

where $as_{\left(i\right)}$ is an asymmetric sector ID, i is a unit-beam sector ID, and M is the total number of unit-beam sectors. In Equation (6), the same multiple values of $as_{\left(i\right)}$ stand for one asymmetric sector, and Algorithm 1 describes the asymmetric sectorization procedure. In this algorithm, M2M GW repeatedly conducts the ‘sector decision’, thereby deciding whether the adjacent unit-beam sectors can be merged or not. M2M GW initializes the variables (*i.e.*, *init_pt*, *decision_index*, *numMergeSector*, *empty_cnt*, *numFrontSector*, and *numRearSector*) that are needed to configure the asymmetric sectors. ‘*init_pt*’ denotes the starting point for each sector decision, which is an ID of the foremost unit-beam sector, and it is updated whenever the sector decision is newly performed. ‘*decision_index*’ indicates the number of times that the sector decision has been performed, and ‘*numMergeSector*’ is the number of unit-beam sectors that can be merged at each sector decision. ‘*empty_cnt*’ is the number of unit-beam sectors with no device (*i.e.*, empty sector). Thus the difference of ‘*decision_index*’ and ‘*empty_cnt*’ indicates the number of asymmetric sectors. Finally, ‘*numFrontSector*’ and ‘*numRearSector*’ indicate the numbers of unit-beam sectors with the same $as_{\left(i\right)}$ from the starting point (*i.e.*, i=1) and from the end point (*i.e.*, i=M), respectively.

The sector decision is iterated for each unit-beam sector to obtain the asymmetric sector ID, $as_{\left(i\right)}$, that has been allocated. First, the empty sector is allocated with $as_{\left(i\right)}=0$. For the unit-beam sectors with devices, M2M GW compares the CR value of each unit-beam sector with *numMergeSector*. At the beginning of the asymmetric sectorization phase, *numMergeSector* is initialized to 1, and it is used as a reference value for the merging of adjacent unit-beam sectors. As a result of each sector decision, M2M GW can merge adjacent unit-beam sectors into one transmission sector referring the number of *numMergeSector*. If all CR values are equal to or larger than *numMergeSector* for the unit-beam sectors from *init_pt* to i, the same $as_{\left(i\right)}$ is allocated to the corresponding unit-beam sectors. Note that $as_{\left(i\right)}$ is obtained as (*decision_index* – *empty_cnt*). The *SectMergeFlag* is used to verify the result of the comparison between the CR value and *numMergeSector*. Therefore, in the case where the CR values for every checked unit-beam sector are equal to or larger than *numMergeSector*, *SectMergeFlag* is set to TRUE and otherwise to FALSE. When all unit-beam sectors are allocated $as_{\left(i\right)}$, M2M GW finally conducts the sector decision for the front and the rear unit-beam sectors to check to merge them. If all CR values for the front and the rear unit-beam sectors are equal to or larger than the sum of *numFrontSector* and *numRearSector*, $as_{\left(i\right)}$ for the rear unit-beam sectors is updated to 1. The unit-beam sectors with the same asymmetric sector ID are merged into one asymmetric sector, and as a result of this algorithm, M2M GW can build up the optimal sectorization for multicast transmission.

**Figure 3:**
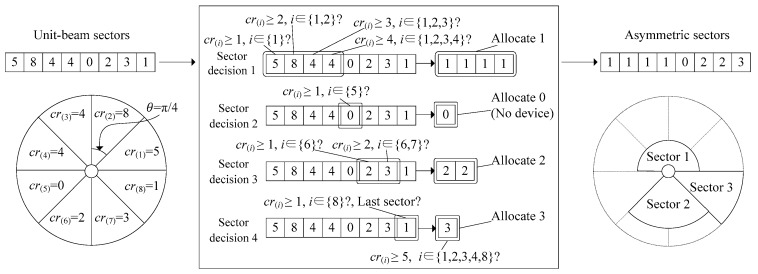
Example of asymmetric sectorization.

Figure 3 shows an operational example of the asymmetric sectorization. In the figure, M2M GW is assumed to use the unit-beam sector with a beamwidth of π/4, which is rotated in an anti-clockwise direction. We also assume that the matrix CR is given by [5,8,4,4,0,2,3,1]. At the 1st unit-beam sector, M2M GW checks $cr_{\left(1\right)}=5$ to see whether it is larger than *numMergeSector* (1). If so, it allocates 1 to $as_{\left(1\right)}$ and increases *numMergeSector* by 1. Then, at the 2nd unit-beam sector, $cr_{\left(1\right)}$ and $cr_{\left(2\right)}$ are compared with the updated *numMergeSector* (2). If both are larger than 2, it also allocates 1 to $as_{\left(2\right)}$. This procedure is continued until $cr_{\left(i\right)}$ is smaller than *numMergeSector*. Accordingly, at the 3rd and 4th unit-beam sectors, both $as_{\left(3\right)}$ and $as_{\left(4\right)}$ are allocated to 1. $as_{\left(5\right)}$ is allocated to 0 (*decision_index* is increased by 1 and *numMergeSector* is initialized to 1) because the 5th unit-beam sector is the empty sector (*i.e.*, $cr_{\left(5\right)}=0$). $as_{\left(6\right)}$ and $as_{\left(7\right)}$ are allocated to 2 considering the empty sector ($cr_{\left(5\right)}=0$). Likewise, $as_{\left(8\right)}$ of the last unit-beam sector gets allocated 3. Finally, M2M GW checks whether the front and the rear sectors can be merged. In our example, this is impossible because $cr_{\left(3\right)}=4$, $cr_{\left(4\right)}=4$, and $cr_{\left(8\right)}=1$ are smaller than 5 (*numFrontSector* + *numRearSector*). Consequently, M2M GW newly builds up three asymmetric sectors, and their beamwidth sizes are π, π/2, and π/4, respectively.
**Algorithm 1** Asymmetric sectorization**INITIALIZE** *init_pt* to 1, *decision_index* to 1, *numMergeSector* to 1, *empty_cnt* to 0, *SectMergeFlag* to TRUE   // variables are initialized**FOR** each unit-beam sector, i, i∈[1,M]   // M is the number of unit-beam sectors   **IF** $cr_{\left(i\right)}$== 0   // unit-beam sector without device     $as_{\left(i\right)}$← 0   // assign zero for asymmetric sector ID    *decision_index* ← *decision_index* + 1   // change temporary sector index    *empty_cnt* ← *empty_cnt* + 1    *init_pt* ← i + 1    *CR_index* ← 0  **ELSE**   // unit-beam sector with device       **FOR** each unit-beam sector ,j, j∈[init_pt,i]         **IF** $cr_{\left(i\right)}$ ≥ *numMergeSector*   // compare each CR value with *numMergeSector*          *SectMergeFlag* ← TRUE        **ELSE**           *SectMergeFlag* ← FALSE           break         **ENDIF**    **ENDFOR**       **IF** *SectMergeFlag* == TRUE          *numMergeSector* ← *numMergeSector* + 1       **ELSE**         *decision_index* ← *decision_index* + 1   // change decision index         *init_pt* ← i         *numMergeSector* ← 2       **ENDIF**         $as_{\left(i\right)}$ ← *decision_index* – *empty_cnt*   // assign value for asymmetric sector ID     **ENDIF**     **IF** $i==M\text{ \&\& }as_{\left(i\right)}!=0$   // current unit-beam sector is the last one      *numFrontSector* ← number of unit-beam sectors where $as_{\left(i\right)}$ is 1      *numRearSector* ← number of unit-beam sectors where $as_{\left(i\right)}$ is (*decision_index* – *empty_cnt*)      **IF** $cr_{\left(i\right)}$ ≥ (*numFrontSector* + *numRearSector*)        $\forall as_{\left(k\right)}$ ← 1, k∈[init_pt,M]   //merge the last sector      **ENDIF**   **ENDIF****ENDFOR**

## 4. Performance Evaluation

We evaluate the performance of the ADM by conducting an experimental simulation. Various performance metrics are considered in this simulation in terms of the number of transmission sectors, transmission time, and aggregate throughput. We compare the results of the simulation to the multicast transmission approach of the IEEE 802.15.3c standard in order to verify the superiority of ADM. In the following subsections, we describe the simulation setting and configuration and discuss the results of the simulation, in detail.

### 4.1. Simulation Setting and Configuration

In the simulation, we consider a conference room scenario, where it is assumed that a number of M2M devices are randomly deployed within the W×L size room and divided into three multicast groups (MGs) (*i.e.*, MG1, MG2, and MG3). The MGs are supposed to be served sequentially from the M2M GW at the center of the room. To support 1080p HD video streaming, we use 1.65 Gbps data rate and a 29.63 μs transmission interval since it requires more than 1.485 Gbps data rate and less than 29.64 μs transmission interval [28]. Considering an HD video streaming application for three MGs, we configure packet of different sizes for each MG, which represents the chunk of data for different video streaming content [29,30,31,32]. We assume that the ADM is implemented on top of the IEEE 802.15.3c PHY layer model and its operational phases are independent with the superframe structure of IEEE 802.15.3c MAC layer. For fair comparison of the network performance, both in ADM and legacy multicast, it is assumed that the capillary M2M network consists of stationary devices, thus the operation of device discovery is performed only once at the time of deployment throughout the whole network lifetime using the unit-beam sectors. However, considering the information types of control messages, we further assume that ADM and legacy multicast use control messages of 50 byte- and 20 byte-size, respectively. The simulation is iterated 50 times for different room sizes, transmission ranges, and beamwidth sizes of the unit-beam sector. The detailed parameters are listed in Table 3.

### 4.2. Simulation Results

**Figure 4:**
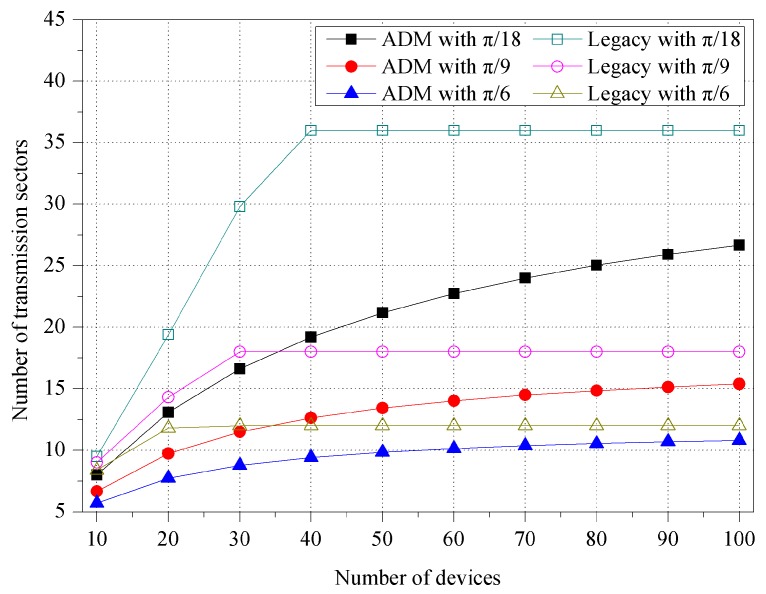
Number of transmission sectors for various unit-beam sectors.

Figure 4 shows the number of transmission sectors for various unit-beam sectors. The number of transmission sectors is the same as repeated directional transmissions for one multicast, and thus it works as system overhead. In the simulation, we use three unit-beam sectors with different beamwidth sizes set to π/18, π/9, and π/6, respectively. The devices are randomly deployed within a transmission range of M2M GW, and the number of devices varies from 10 to 100. In ADM, the number of sectors slowly increases as the number of devices increases. ADM tries to maintain the minimum number of sectors by merging the adjacent unit-beam sectors in accordance with the location of the devices. On the other hand, for the legacy multicast, this sharply increases until reaching the number of unit-beam sectors of the initial simulation settings. Specifically, for one multicast transmission, ADM maintains 34.84%, 23.59%, and 19.06% fewer sectors than legacy multicast on average, when the beamwidths of unit-beam sector are π/18, π/9, and π/6, respectively.

**Figure 5:**
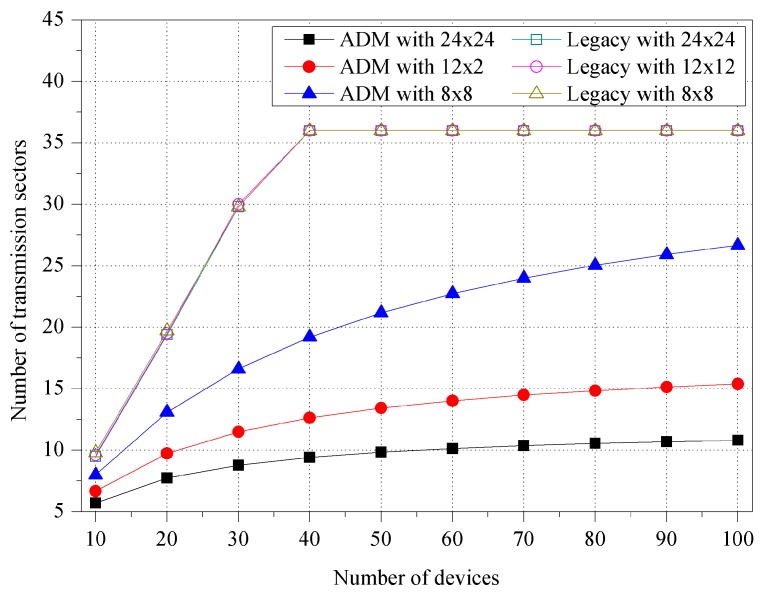
Number of transmission sectors for various room sizes.

Figure 5 shows the impact of the room size. We assume three square-shaped conference room environments with different areas of 24 × 24 m^2^, 12 × 12 m^2^, and 8 × 8 m^2^, respectively. The M2M GW is placed in the center of the room, and it uses the unit-beam sector with π/18 beamwidth. In the room with a size of 8 × 8 m^2^, the ADM exhibits the minimum number of sectors since it can use the asymmetric sectors with the widest bandwidth to cover the room. Specifically, the average number of sectors for ADM is 19.23 (in 24 × 24 m^2^), 12.23 (in 12 × 12 m^2^), and 8.93 (in 8 × 8 m^2^), respectively. However, the legacy multicast maintains almost the same number of sectors regardless of the room size, and in each room environment, the ADM exhibits approximately 38.10%, 60.97%, and 71.30% less number of sectors compared to the legacy multicast.

**Figure 6:**
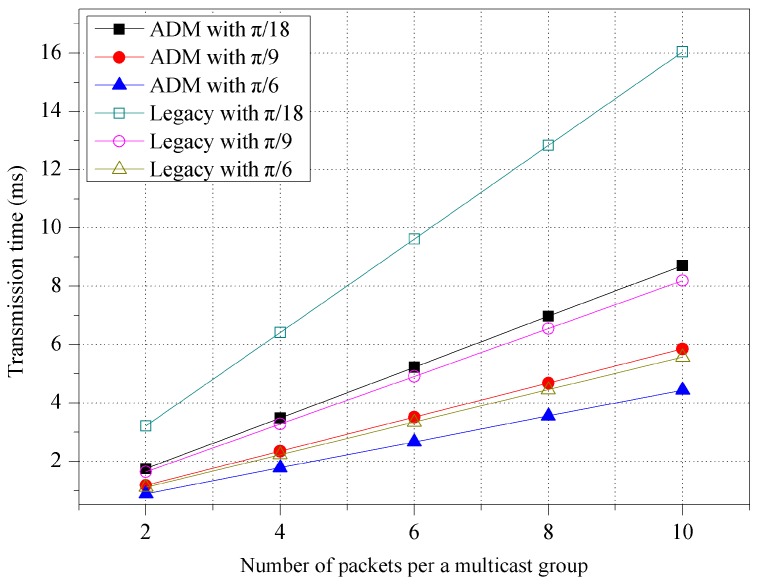
Transmission time for various unit-beam sectors.

Figure 6 shows the transmission time for various beamwidth sizes of the unit-beam sector. The number of packets per a multicast group in the x-axis means the number of chunks that are continuously transmitted in one transmission. As in Figure 5, the unit-beam sector of the M2M GW has beamwidths of π/18, π/9, and π/6, respectively, to provide an appropriate transmission range according to each room size (*i.e.*, 24 × 24 m^2^, 12 × 12 m^2^, and 8 × 8 m^2^). In the figure, the use of a small beamwidth increases the transmission time due to more sector switchings (*i.e.*, a larger number of transmissions) to complete one multicast. Specifically, when the number of packets is 6, the transmission time for ADM decreases from 5.23 ms (beamwidth of π/18) to 2.66 ms (beamwidth of π/6). When compared to legacy multicast, ADM exhibits an approximately 31% better performance in terms of the transmission time.

**Figure 7:**
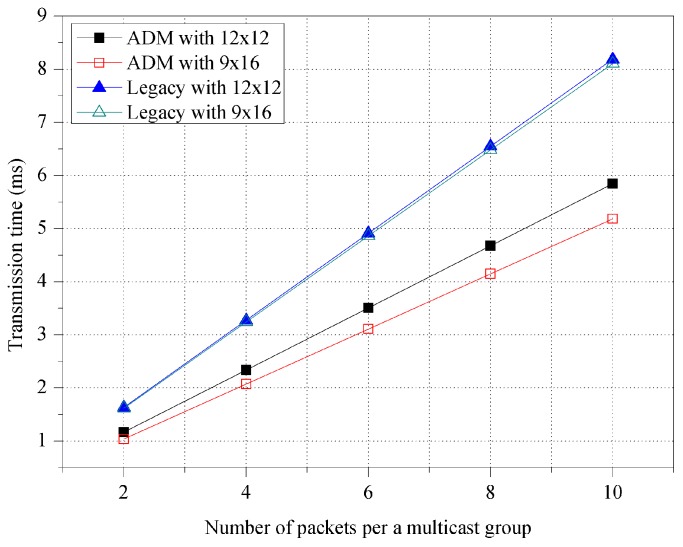
Transmission time for various room shapes.

Figure 7 shows the transmission time for various room shapes. The network performance of ADM is affected by the shape of the room while that of legacy multicast is not. In a rectangular room (*i.e.*, 9 × 16 m^2^), ADM exhibits a transmission time that is 11.31% smaller than that in a square room (*i.e.*, 12 × 12 m^2^). The devices in the rectangular room tend to be placed near the M2M GW, and ADM is thus likely to maintain a smaller number of sectors with a large beamwidth. On the other hand, for legacy multicast, M2M GW has to maintain the same number of sectors regardless of the room shape. The results of the simulation show that ADM obtains a transmission time that is 32.30% smaller than that of legacy multicast in a rectangular room. Considering that most conference rooms are rectangular, ADM could therefore be useful for real environments.

We evaluate the aggregate throughput, which can be estimated as follows [33].

(7) $$S=\frac{E[P]\sum_{i=1}^{N}n_{i}}{\sum_{i=1}^{N}E[T_{i}]}$$

where N is the number of sectors, $n_{i}$ is the number of devices within the sector i, E[P] is an average length of the packet payload, and $E[T_{i}]$ is the average transmission time at sector i. Regarding the evaluation of the aggregate throughput, we consider various experimental factors, such as the beamwidth size of the unit-beam sector, the shape of room, and the position of M2M GW.

**Figure 8:**
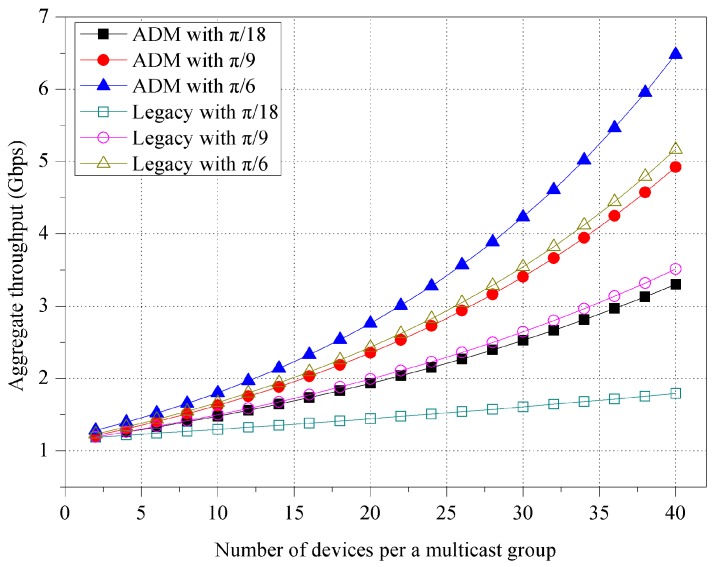
Aggregate throughput for various unit-beam sectors.

First of all, Figure 8 shows the impact of the beamwidth size of unit-beam sector. In both ADM and legacy multicast, the aggregate throughput increases as the beamwidth size of unit-beam sector increases due to the reduced switching delay of the directional transmission. With asymmetric sectorization under the same environment, ADM achieves approximately 19.91% higher aggregate throughput compared with legacy multicast.

**Figure 9:**
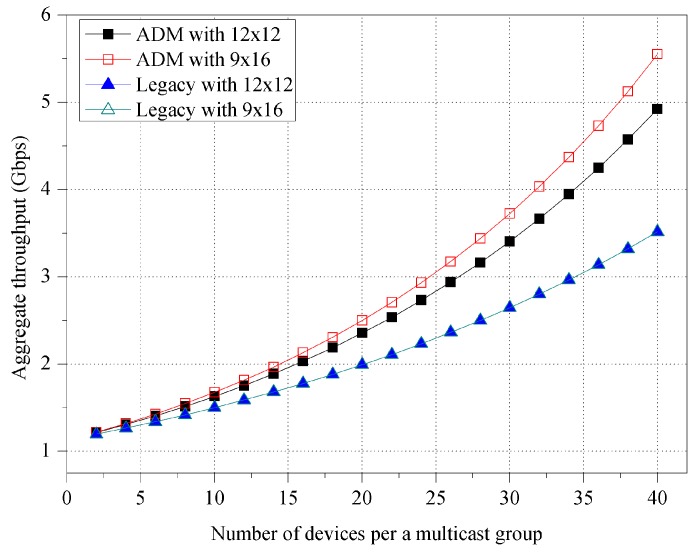
Aggregate throughput for various room shapes.

Figure 9 shows the impact of the room shape on the aggregate throughput. In the simulation, the beamwidth of the unit-beam sector is set to π/9. As seen in Figure 7, ADM exhibits a shorter transmission time in a room with a rectangular shape and likewise obtains an improved throughput performance. In the figure, ADM exhibits 7.40% higher throughput on average when compared to the case of a square room. However, the throughput of legacy multicast is not affected by the room shape.

**Figure 10:**
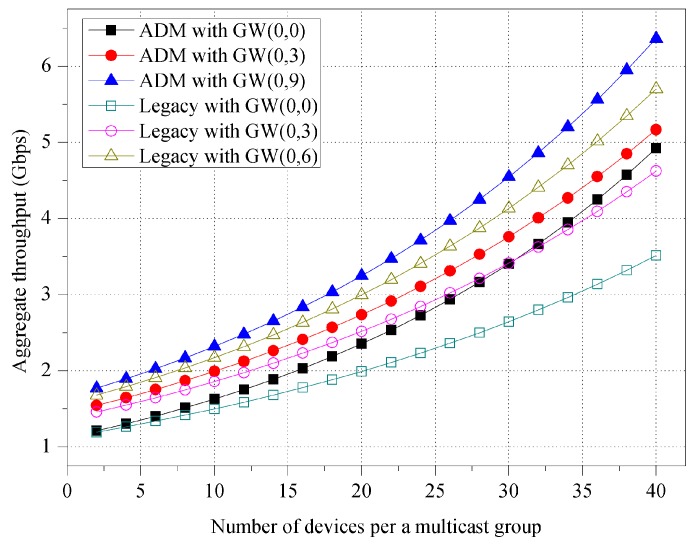
Aggregate throughput for various positions of M2M GW.

As mentioned earlier, the sectorization of ADM is based on the distance of each device from the M2M GW, and it is thus necessary to evaluate the impact of the position of the M2M GW in the conference room. In Figure 10, we measure the aggregate throughput for various positions of M2M GW. M2M GW is initially placed in the center (*i.e.*, GW(0, 0)) of a 12 × 12 m^2^ room, and it then moves upward by 3 m (*i.e.*, GW(0, 3)) and 6 m (*i.e.*, GW(0, 6)). The beamwidth of the unit-beam sector is set to π/9. In both the ADM and legacy multicast, the aggregate throughput increases as the M2M GW moves from the center. When M2M GW is located at the biased position in the room, the number of empty sectors increases, and the other sectors are likely to include more devices. In the simulation, we configure the M2M GW to not transmit any packet at the empty sector with no device. Thus, they commonly exhibit higher throughput performance in the biased position. Specifically, the ADM shows a throughput that is higher by 11.53% on average when compared with legacy multicast as a result of the difference in the number of sectors.

We investigate the control overhead of ADM. The operation of the device discovery incurs the control overhead due to control messages (*i.e.*, join-request and response messages) that are exchanged in a carrier sense multiple access with collision avoidance (CSMA/CA) manner. This overhead problem is common for both ADM and legacy multicast. This is because the same unit-beam sector is used in the device discovery phase, thus the same numbers of control messages are required. However, each mechanism uses a control message of a different size (*i.e.*, ADM: 50 bytes, legacy multicast: 20 bytes), thereby mainly causing the difference of control overhead between ADM and legacy multicast.

**Figure 11:**
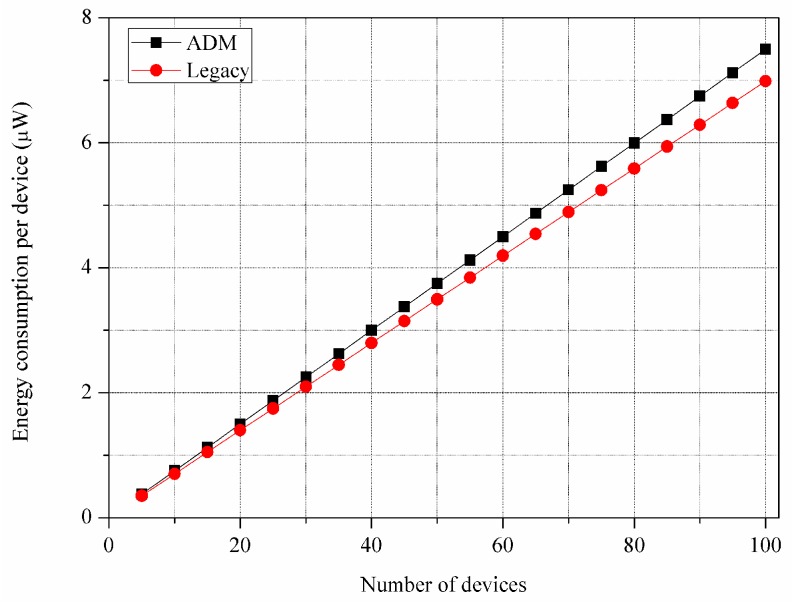
Energy consumption per device for control message exchange.

**Figure 12:**
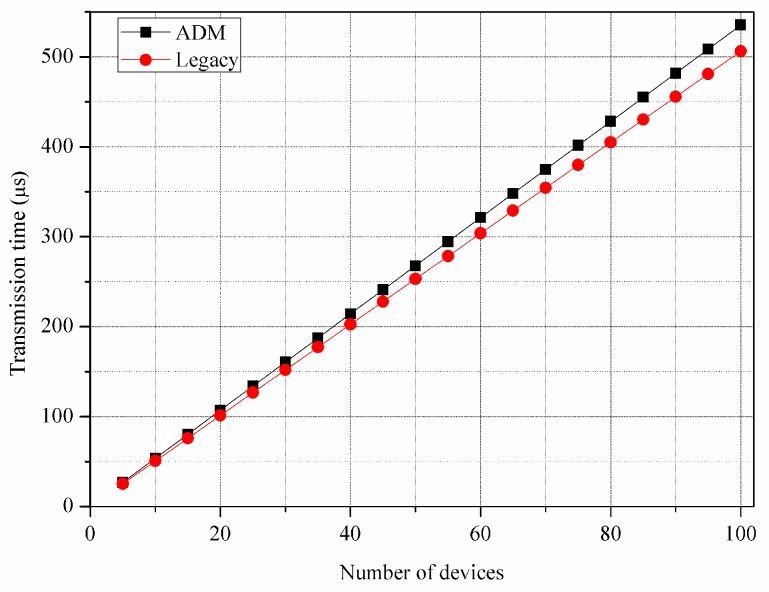
Transmission time for control message exchange.

Figure 11 and Figure 12 show the control overhead for the control message exchange with respect to the energy consumption per a device and the transmission time, respectively. In the figures, we consider the overhead only for the transmission and reception of the control messages. The ADM exhibits 6.79% higher energy consumption and 5.43% longer transmission time compared to the legacy multicast. Table 4 shows the control overhead of the entire device discovery phase including the channel access operation of CSMA/CA, where the ADM exhibits much smaller difference of control overhead compared to the previous results. Specifically, ADM exhibits 0.89% higher energy consumption and 0.78% longer transmission time. This is because the impact of difference on the control message sizes is reduced due to the CSMA/CA backoff delay. This overhead problem can be ignored over a long period of network operation when considering that the device discovery is performed only once at the time of deployment throughout the whole network lifetime.

**Figure 13:**
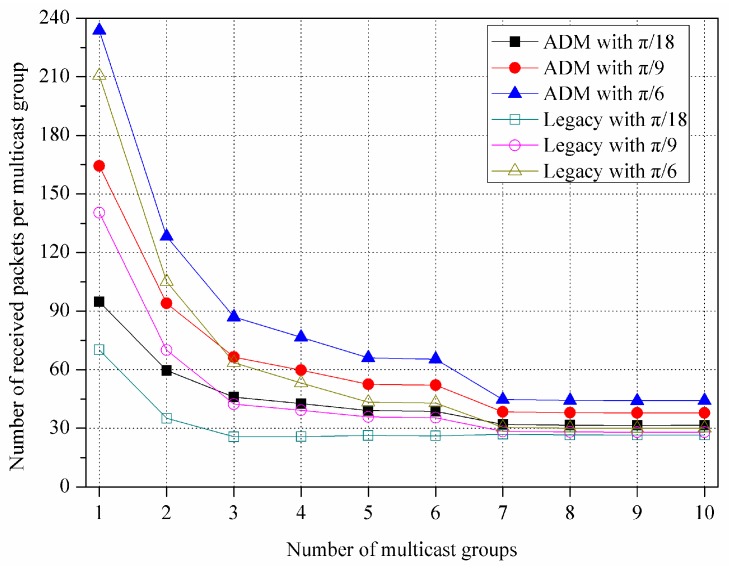
Number of received packets per multicast group.

**Figure 14:**
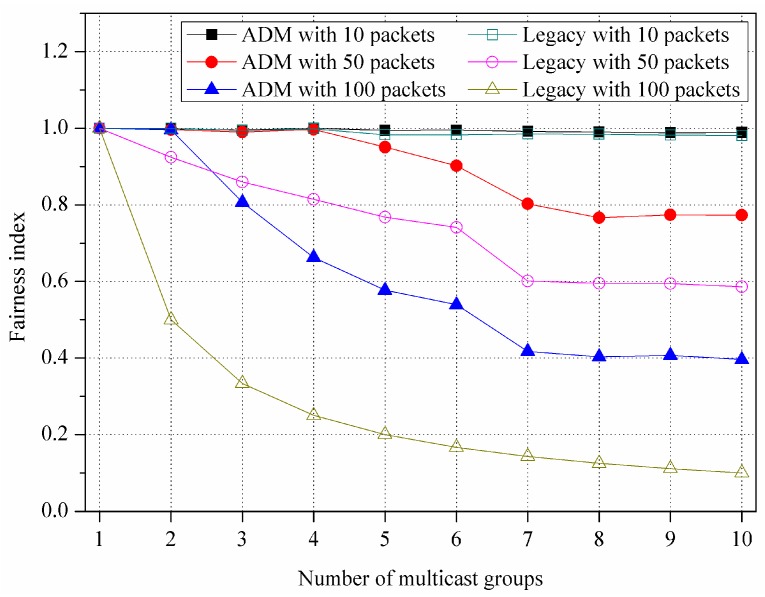
Variation of fairness index for various numbers of packets per multicast group.

In Figure 13 and Figure 14, to investigate the effect of the variation on the number of MGs, we consider the new simulation environments, where 100 devices are randomly deployed within 10 × 10 m^2^ size room and they compose multiple MGs. The M2M GW transmits data packets of 4 Kbytes to each MG during the simulation time of 100 ms.

Figure 13 shows the number of received packets per an MG for various unit-beam sectors. In this experiment, we assume that M2M GW transmits 10 packets to each MG sequentially. In both ADM and legacy multicast, the number of received packets per an MG decreases as the number of MGs increases. This is because a larger number of MGs are served from the M2M GW for the same simulation time (*i.e.*, 100 ms). In the figure, the devices running the ADM receive more packets than the legacy multicast because the ADM builds up a smaller number of transmission sectors by merging the adjacent unit-beam sectors via an asymmetric sectorization procedure, while the legacy multicast uses only the unit-beam sectors. Specifically, when the beamwidth of unit-beam sector is set to π/18, π/9, and π/6, the ADM exhibits approximately 29.42%, 25.75%, and 23.36% higher reception ratio of packets compared to the legacy multicast, respectively. Note that, in our simulated scenario, when the number of MGs is more than seven, it maintains constant. When the number of MGs increases, the average number of devices (*i.e.*, MG members) per an MG is reduced, thereby also reducing the effect of the asymmetric sectorization. Thus, the number of received packets per an MG is no longer declining. Moreover, for the same reason, its difference between ADM and legacy multicast decreases as the number of MGs increases.

Figure 14 shows the fairness index for various numbers of packets per an MG. As mentioned earlier in Section 4.1, the number of packets per an MG means the number of chunks that are continuously transmitted in one transmission under HD video streaming application scenario, in which it is set to 10, 50, and 100, respectively. The fairness index (*F*) can be calculated as follows [34]:

(8) $$F=\frac{{\left(\sum_{i=1}^{n}x_{i}\right)}^{2}}{n\sum_{i=1}^{n}x_{i}{}^{2}}$$

where n is the number of MGs, i is the MG ID, and $x_{i}$ is the fairness parameter, which means the number of packets that each MG receives. The fairness index of ADM starts to decrease at a larger number of MGs and keeps a higher value compared to that of legacy multicast, due to the benefit of asymmetric sectorization. Specifically, when the number of packets per an MG is 50 and 100, the ADM exhibits approximately 16.39% and 52.79% higher fairness index compared to that of the legacy multicast, respectively. Meanwhile, in the case that the number of packets per an MG is 10, both of the ADM and the legacy multicast can almost evenly serve all the MGs within the limited simulation time of 100 ms, thus their fairness indices retain the value of one.

## 5. Conclusions 

This paper presents asymmetric directional multicast transmission (ADM) for capillary M2M using mmWave communications. The optimum sectorization for a deployment pattern of multicast group members is achieved with ADM by merging the unit-beam sectors indicating the position of each M2M device. Thus, the minimum numbers of asymmetric sectors are built. The performance was evaluated by conducting experimental simulations with various factors that affect the network performance, such as variations in the beamwidth size of the unit-beam sector, the shape of room, and the position of M2M GW. The results of the simulation show that ADM can achieve considerable improvement in terms of the number of transmission sectors, transmission time, and aggregate throughput. In particular, ADM can be useful in real environments since it is shown to achieve a high performance in various room shapes and in different positions of M2M GW.

In our ongoing and future work, we plan to design the transmission scheduling strategy of ADM under the IEEE 802.15.3c MAC superframe environment and the optimization of the contention access period (CAP) duration for the system configuration will be discussed. Moreover, for future work, the theoretical analysis based on the mathematical modeling will be given to verify the effectiveness of this extended work and the accuracy of the simulations.

## Figures and Tables

**Table 1:** Example of the maximum transmission range of the directional antennas.

MCS	Sensitivity (dB)	BeamWidth (rad)	Maximum Transmission Range (m)
Data rate: 412 Mbps Modulation: BPSK Spreading factor: 4 FEC type: RS(255,239)	-61	π/18	60.47
		π/9	30.23
		π/6	15.11
		π/3	10.07
		π	3.35
Data rate: 825 Mbps Modulation: BPSK Spreading factor: 2 FEC type: RS(255,239)	-58	π/18	42.81
		π/9	21.40
		π/6	14.27
		π/3	7.13
		π	2.37
Data rate: 1650 Mbps Modulation: BPSK Spreading factor: 1 FEC type: RS(255,239)	-55	π/18	30.30
		π/9	15.15
		π/6	10.10
		π/3	5.05
		π	1.68

**Table 2:** MCS categorization of mmWave SC-PHY.

MCS Class	MCS ID	Data Rate (Mbps)
Class 1	0	25.8
	1	412
	2	825
	3	1650
	4	1320
	5	440
	6	880
Class 2	7	1760
	8	2640
	9	3080
	10	3290
	11	3300
Class 3	12	3960
	13	5280

**Table 3:** Simulation parameters.

Parameter	Value	Parameter	Value
PHY	IEEE 802.15.3c	Antenna	Sectored antenna
Traffic application	CBR	Slot time	20 μs
Beamwidth of unit-beam sector	π/18, π/9, π/6	SIFS	2.5 μs
MG1 packet size	2 Kbytes	Preamble duration	8.157 μs
MG2 packet size	3 Kbytes	Transmission power	20 mW
MG3 packet size	4 Kbytes	Receive power	15 mW
Data rate	1.65 Gbps	Idle power	10 mW
Sensitivity	-55 dBm	Guard time	0.02 μs

**Table 4:** Control overhead of device discovery.

Control Overhead	ADM	Legacy
Average energy consumption (μW)	117.01	115.98
Average transmission time (μs)	11.59	11.49
